# Supplementary material for: Comparison of neoadjuvant treatment and surgery first for resectable or borderline resectable pancreatic carcinoma: A systematic review and network meta-analysis of randomized controlled trials
Source: PLoS One. 2024 Mar 7;19(3):e0295983. doi: 10.1371/journal.pone.0295983 (PMC10919605; doi:10.1371/journal.pone.0295983)
Supplement: Attachment — Submitted filename: Response to Reviewers.docx [file pone.0295983.s002.docx]

Dear Editor:

We would like to resubmit the revised manuscript (entitled “Comparison of neoadjuvant treatment and surgery first for resectable or borderline resectable pancreatic carcinoma: a systematic review and network meta-analysis of randomized controlled trials”, PONE-D-23-23777) for consideration by PLOS ONE. We would like to thank the reviewer for thoroughly reviewing our manuscript and making many thoughtful comments. We were very pleased to see the reviewer recognized the novelty and potential significance of our work. We have revised and added some parts, described in detail below, and revised the manuscript to address reviewers’ comments.In this revised version,changes to our manuscript were all highlighted within the document by using red colored text and here are our point-by-point responses:

**Reviewer #1:** Thank you for your nice comments on our article. According to your suggestions, we have make clear and corrected several mistakes in our previous draft. We have made extensive revisions to our previous draft. The detailed point-by-point responses are listed below.

**Comment 1:** Given the right clarification on stereotactic radiotherapy in the "Discussion" (page 15, line 5), it could be made explicit that the chemoradiotherapy referred to conventional radiotherapy using standard dose.

Answer: Thanks for your suggestion. We have made a clear definition on stereotactic radiotherapy^1^ in our previous article and the correctional parts were highlighted by using red colored text. The correctional limitation was added in Page15 row 5-6.
 **Comment 2:** In the "Conclusions", given the results of the systematic review conducted, in addition to the impact on the prognosis, it should be specified that the type of NT with the best impact on R0 after surgery is chemoradiotherapy, (otherwise, in my opinion, an important result could be eclipsed).

Answer: Thanks for your suggestion. We have refined our conclusions better and the enhancive parts were highlighted by using red colored text. The enhancive limitation was added in Page19 row 10- 11.
 **Comment 3:** On page 9 line 12 "To" must be corrected to "o"

Answer: Thanks for your suggestion. We have corrected the mistake on page 9 line 12.

Reviewer #2: Thank you for your valuable comments on our article. According to your suggestions, we have supplemented several parts to explain and corrected several mistakes in our previous draft. We have made extensive revisions to our previous draft. The detailed point-by-point responses are listed below.

**Comment 1:** This sentence is generic and unclear: " Compared with surgery first, neoadjuvant therapy did increase the R0 resection rate (RR = 1.53, I2 = 0%, P＜ 0.00001), there was a certain possibility that gemcitabine + cisplatin (Gem+Cis) +Radiotherapy was the most favorable in terms of the fact that there was no significant difference concerning the results from the individual studies." Also this sentence is unclear : "Furthermore, most published meta analyses reported higher R0 resection rates overall with neoadjuvant therapy, contradicting the clear benefit of neoadjuvant therapy"

Answer: Thanks for your suggestion. We make some optimizations to render these sentence more clear.

Sentence from the first draft was changed to "Compared with surgery first, neoadjuvant therapy did increase the R0 resection rate (RR = 1.53, I^2^ = 0%, P＜ 0.00001), besides, by the net-work there was a certain possibility that gemcitabine + cisplatin (Gem+Cis) + Radiotherapy was the most favorable. " In Page 4 row 9- 10

Also, another sentence from the first draft was changed to "Furthermore, most published meta-analyses^2-4^ reported higher R0 resection rates but a lower surgical resection rate overall with neoadjuvant therapy, contradicting the clear benefit of neoadjuvant therapy." In Page 5 row 12- 13 and in Page 6 row 1
 **Comment 2:** As a radiation oncologist, I believe that the results are a little bit inconclusive: radiotherapy increases R0, this is a somewhat expected result, but does it improve survival? Do chemotherapy-only studies have better survival outcomes? Eventually it would be worth underlining in the conclusions that, just as the studies using adjuvant gemcitabine as monotherapy are outdated, so too are the studies using conformal radiotherapy techniques, furthermore, the doses and times of radiotherapy can be an influencing factor.

Answer: Thanks for your valuable suggestions. Based on studies those met our inclusion criteria, we considered that neoadjuvant chemoradiotherapy could increase R0, and by Bayesian network gemcitabine + cisplatin (Gem+Cis) + Radiotherapy had a higher likelihood of being the most favorable regimen. Similarly, we considered neoadjuvant chemoradiotherapy could improve mOS, but by Bayesian network Cisplatin/ Epirubicin then Gemcitabine/ Capecitabine (PEXG) might be relative best. Finally, as your valuable suggestions, outdated chemotherapy region and different radiation doses could make an influence on our results. We incorporated these deficiencies in our limitations and conclusion. In Page 19 row 1, 5- 6, 12- 13 and Page 20 row 1
 **Comment 3:** I am not an expert in statistics so tables 2 and 3 are a little difficult for me to understand, perhaps a better explanation would be needed.

Answer: Thanks for your valuable suggestions. We have deleted some repetitive expression to make the data clearer and more concise. And we have re-uploaded tables 2 and 3, also a more detailed explanation have been added in tables.

Thank you very much for your valuable comment.We had noticed these problems you mentioned above before we conceived of this study.We hope that the revised version of the manuscript is now acceptable for publication in your journal.

I look forwar to hearing from you.

With best wishes,

Your sincerely,

Yang Cao.

**Reference**

1. Katz, M. H. G. *et al.* Efficacy of Preoperative mFOLFIRINOX vs mFOLFIRINOX Plus Hypofractionated Radiotherapy for Borderline Resectable Adenocarcinoma of the Pancreas: The A021501 Phase 2 Randomized Clinical Trial. *JAMA oncology* **8**, 1263-1270, doi:10.1001/jamaoncol.2022.2319 (2022).
2. van Dam, J. L. *et al.* Neoadjuvant therapy or upfront surgery for resectable and borderline resectable pancreatic cancer: A meta-analysis of randomised controlled trials. *European journal of cancer (Oxford, England : 1990)* **160**, 140-149, doi:10.1016/j.ejca.2021.10.023 (2022).
3. Cloyd, J. M. *et al.* Neoadjuvant Therapy for Resectable and Borderline Resectable Pancreatic Cancer: A Meta-Analysis of Randomized Controlled Trials. *Journal of clinical medicine* **9**, doi:10.3390/jcm9041129 (2020).
4. Ye, M. *et al.* Neoadjuvant chemotherapy for primary resectable pancreatic cancer: a systematic review and meta-analysis. *HPB : the official journal of the International Hepato Pancreato Biliary Association* **22**, 821-832, doi:10.1016/j.hpb.2020.01.001 (2020).
